# Microwave technology for detecting traumatic chest injuries in a porcine model

**DOI:** 10.1007/s11517-025-03495-8

**Published:** 2025-12-23

**Authors:** Philipp Seidel, Nils Petter Oveland, Marianne Oropeza‐Moe, Linh Nguyen, Andreas Fhager, Mikael Persson, Mikael Elam, Stefan Candefjord

**Affiliations:** 1 Department of Intensive Care Medicine, Stavanger University Hospital, Stavanger, Norway; 2 Department of Quality and Health Technology, Faculty of Health Sciences, University of Stavanger, Stavanger, Norway; 3 Department of Anesthesiology, Stavanger University Hospital, Stavanger, Norway; 4 Department of Production Animal Clinical Sciences, Faculty of Veterinary Medicine, Norwegian University of Life Sciences, Sandnes, Norway; 5 Department of Electrical Engineering, Chalmers University of Technology, Gothenburg, Sweden; 6 Department of Clinical Neurophysiology, Sahlgrenska University Hospital, Gothenburg, Sweden; 7 SAFER Vehicle and Traffic Safety Centre at Chalmers, Gothenburg, Sweden

**Keywords:** Microwave technology, Traumatic chest injury, Haemothorax, Pneumothorax, Haemopneumothorax, Porcine model, Point-of-care diagnostic

## Abstract

The electromagnetic properties of microwaves are altered by the dielectric properties of biological tissues, allowing microwave technology (MWT) to be used to detect free air and blood in the body. This animal study investigated whether MWT could be used as a point-of-care approach for the diagnosis of pneumothorax (PTX), haemothorax (HTX) and haemopneumothorax (HPTX). A porcine model of PTX (50–1500 mL), HTX (50–750 mL) and HPTX (750 mL blood and 50–1500 mL air) was established, by injecting air and blood in the pleural cavity. A MWT device with eight antennas was positioned around the chest, and all antenna pair combinations were measured and analysed for attenuation of magnitude. All stages of HPTX and larger stages of PTX and HTX induced significant changes in the microwave signals. Attenuation of the signal increased with increasing air and/or blood volume. Although trauma-induced changes were significant compared to the baseline, the interindividual variance exceeded the trauma-related changes. Microwave signals were significantly altered by free air and blood and the attenuation correlated with the size of the PTX, HTX and HPTX. MWT can potentially complement point-of-care diagnostics for severe thoracic injuries. However, further improvement is necessary before clinical testing.

**Graphical abstract:**

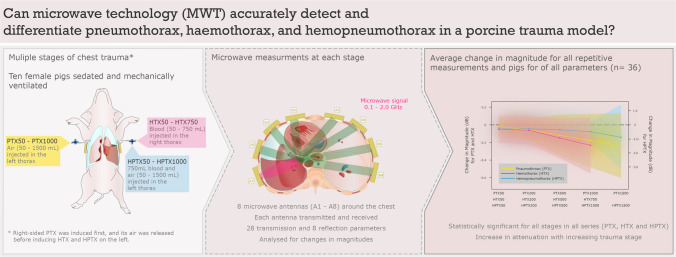

**Supplementary Information:**

The online version contains supplementary material available at 10.1007/s11517-025-03495-8.

## Background

Traumatic injury is a major cause of death in the younger population worldwide [1, 2]. Thoracic trauma is directly or indirectly responsible for approximately 50% of trauma-related deaths and can be caused by blunt or penetrating trauma [3]. Severe chest injuries pose a significant risk not only to civilians but also to military personnel, with preventable deaths reaching up to 80% for uncontrolled bleeding [4–6]. Thoracic trauma, whether in civilian or combat settings, can cause blood (i.e., haemothorax, HTX), air (pneumothorax, PTX) or both (haemopneumothorax, HPTX) to accumulate in the pleural cavity [7]. These injuries are often associated with on-scene patient instability of severe hypotension and hypoxemia, where a failure to diagnose and treat rapidly may cause death. Despite PTX, HTX and HPTX being severe injuries, they can be challenging to diagnose, particularly in the prehospital setting [8, 9].

The standard approach for detecting traumatic HTX and/or PTX is plain chest X-ray (CXR) imaging. Injured patients are often confined to the supine position for neuraxial protection, causing blood to accumulate posteriorly and air anteromedially to the lungs [10–12]. Blood trapped behind or air in front of the lung is particularly difficult to detect and quantify on supine CXRs, and even large amounts of blood and air may be overlooked during hospitalization. In the supine position, HTX up to a volume of 1000 mL can be missed by CXR [13]. Therefore, computed tomography (CT) scans are the gold standard and can even detect very low volumes of free blood in the thorax [14]. The same diagnostic challenges have been reported for traumatic PTX [13].

Physicians are challenged with a diagnostic dilemma when encountering trauma patients because physical examination, including anteroposterior supine CXR, can be insufficient for diagnosing severe thoracic injuries. An urgent CT scan can solve the diagnostic dilemma, but in several settings, such as the prehospital, battlefield and remote settings, patients do not have access to these diagnostic modalities, including CXR. Ultrasound (US) is the only available diagnostic modality in addition to clinical examination in these situations. Today, US machines are battery-powered, hand-held, and high-quality diagnostic tools that can be used anywhere. Although point-of-care US is the most versatile diagnostic adjunct in the prehospital setting, it requires extensive training and is highly operator dependent. The sensitivity and specificity for detecting PTX are approximately 69% and 99%, respectively [15], and those for detecting HTX are 89% and 69%, respectively [16]. Continuous monitoring is not possible with either US or, where available, x-ray (CXR, CT). Discontinuous monitoring through repeated recordings has all the disadvantages mentioned for each recording and is almost impossible in transport situations. Thus, new alternative methods to diagnose chest trauma are needed for civilian and military prehospital settings, as well as in other situations that require acute bedside diagnosis and monitoring of thoracic injuries.

Microwave technology (MWT) fulfils many of the requirements of an ideal point-of-care test. It is noninvasive, nonradiating, rapid, mobile, and repeatable at the patient’s bedside, offering the possibility of continuous monitoring. This technology has been used to diagnose intracranial injuries after trauma and stroke [17–19] and has shown potential in the diagnosis of internal injuries in the torso and abdomen [20–22]. In addition to its applications in the field of trauma, microwave technology has also been explored for other thoracic applications, such as non-invasive monitoring of vital signs and respiratory parameters [23, 24].

The advantages of MWT over current imaging and detection technology lie in the physics of electromagnetic waves and how they propagate through the body. The waves easily penetrate human tissue, including dense structures such as bone, with no reported side effects, no radiation exposure, and robustness to shadowing [18]. Free air or blood in body cavities may cause altered propagation and attenuation of the microwave signal. These changes can be measured and analysed.

In this study, we evaluated the use of MWT for the assessment of traumatic thorax injuries in a porcine model with isolated PTX, isolated HTX and combined HPTX.

## Methods

This study was part of a series of experiments to evaluate MWT as a diagnostic tool for both thoracic and abdominal injuries. The various experiments investigating PTX, HTX and HPTX were carried out in the same experimental model as previously described by Candefjord et al. [22] The combination of thoracic and abdominal injuries in the same laboratory animal models reduced the number of animals needed for this study. This is in line with the 3R Principles, with the objective of avoiding animal experiments altogether (Replacement) and limiting the number of animals used (Reduction) and their suffering (Refinement) in tests to an absolute minimum. Importantly, the thoracic part of the experiment was not affected by the abdominal bleeding, as injuries to the abdomen were introduced after the completion of the chest MWT measurements [22].

### Ethical considerations

All experiments performed in this animal model were reported in accordance with the ARRIVE guidelines. Ethical approval for the experiments was granted by the Norwegian Food Safety Authority (approval no. 11933). All procedures were carried out at the Norwegian University of Life Sciences in Sandnes, Norway, and conformed to the Norwegian Animal Welfare Act (LOV-2009–06–19–97), the Regulation Concerning the Use of Animals for Scientific Purposes (FOR-2015–06–18–761) and the Norwegian Regulations on Swine Husbandry (FOR-2003–02–18–175).

### Animal model and anaesthesia

The number of pigs included in this study was based on a priori experience with similar studies, and the sample size was considered sufficient for a proof-of-concept study. The inclusion criteria were female, hybrid, clinically healthy finisher pigs from a selected farm at Jaeren in Norway. The animals had not been subjected to any experiments previously. Additionally, the pigs had to be within the weight range of 55 kg to 70 kg bodyweight. No animals were excluded (exclusion criteria: animals showing any signs of reduced general health, such as increased body temperature above 39.0 degrees Celsius, increased respiratory rates, reduced feed intake or clinical signs of pain, such as back arching, stiff gait, lameness, prolonged vocalization, or trembling). No randomization of the animals was necessary. The animals were kept in the same environment (same pen in groups of 3 to 4 animals), and anaesthesia was induced in a separate pen. The environmental temperature was set to 18 degrees Celsius, according to recommendations for this particular size of pig. Animals were selected from each pen successively so that disturbances in group dynamics within each pen were equal when one individual was removed.

Ten female hybrid finisher pigs (Durox x Landrace/Yorkshire, DDYL) with an average age of 16 weeks and a body weight of 63.7 ± 5.0 kg were sedated with intramuscular midazolam (1 mg/kg, Midazolam 5 mg/mL, B. Braun, Germany) and ketamine (15 mg/kg, Ketador vet. 100 mg/mL, Richter Pharma AG, Wels, Austria).

After sedation, intravenous access was established, and anaesthesia was induced by 160 mg/h propofol (Propofol-Lipuro, 10 mg/mL, B. Braun, Germany), 300 µg/h fentanyl (Fentanyl 50 µg/mL, Hameln, Germany) and preintubation boluses of 50–100 mg ketamine. After intubation, the animals were ventilated with positive pressure ventilation in volume-controlled mode (Vt 400–600 mL, 16–18 bpm), and the minute volume was adjusted to maintain the etCO2 within the normal range (4.0–6.5 kPa). The FiO2 was set to 0.3. Maintenance of anaesthesia was achieved by intravenous infusion of 100–150 mg/h propofol and 300–600 µg/h fentanyl. The total amount of maintenance fluid given was 1000 mL of crystalloids. All animals were monitored (Monitor Agilent, V24C, M1205A, Agilent, Boeblingen, Germany) by electrocardiography, deep intranasal temperature, oxygen saturation, end-tidal carbon dioxide and invasive blood pressure. A single lumen catheter (Certofix Mono, B. Braun, Germany) was placed in the femoral arteria to measure invasive blood pressure and to draw blood to simulate thoracic cavity bleeding (i.e., HTX and HPTX). All measured vital parameters were recorded at five-minute intervals. The experiment was terminated with an injection of 140 mg/kg pentobarbital (Exagon®vet, 400 mg/mL, Richter Pharma AG, Wels, Austria) while deep anaesthesia continued.

### Porcine chest injury model

The porcine model described by Oveland et al. [25, 26] was modified to first simulate a right-sided PTX, then a left-sided HTX and finally a left-sided combination of both blood and air (i.e., HPTX). The experimental unit was a single animal, and individual pigs were used as their own controls. The first experimental series was initiated with the insertion of a three-way stopcock catheter (BD Connecta, 10 cm, BD Medical, USA) into the pleural space through a mini-thoracotomy on the anterior right hemithorax. The surgical incision was closed by subcutaneous and cutaneous stitches, with the catheter anchored to the surrounding muscles and skin. To simulate a progredient PTX, air was induced by consecutive injections into the pleural cavity using a 50 mL syringe (Omnifix, 50 mL, B. Braun Medical, Germany). The intrapleural air volume was increased in five incremental steps from 50 to 1500 mL, as shown in Table 1. Microwave measurements were performed at baseline after catheter insertion and then after each air injection. To avoid haemodynamic instability, the three-way stopcock catheter was opened on the right side to release any excessive air pressure (i.e., tension PTX) before commencing on the left side.

**Table 1:** Stages of pneumothorax, haemothorax and haemopneumothorax

Series 1		Series 2		Series 3		
Pneumothorax right side		Haemothorax left side		Haemopneumothorax left side		
Stage	Volume of air	Stage	Volume of blood	Stage	Volume of blood	Volume of air
Baseline*	0 mL	Baseline*	0 mL			
PTX50	50 mL	HTX50	50 mL	HPTX50	750 mL	50 mL
PTX200	200 mL	HTX200	200 mL	HPTX200	750 mL	200 mL
PTX500	500 mL	HTX500	500 mL	HPTX500	750 mL	500 mL
PTX1000	1000 mL	HTX750	750 mL	HPTX1000	750 mL	1000 mL
PTX1500	1500 mL			HPTX1500	750 mL	1500 mL^#^

The various stages of PTX (right side), HTX and HPTX (both left side) with respective total amount of air and blood in the pleural cavities. The HPTX was induced with incremental volumes of air injected on top of a fixed volume of 750 mL of blood

*Baseline measurements after catheter insertion, but before injection of air or blood

# Insertion of 1500 mL of air was stopped in haemodynamically unstable animals. All measurements before this stage were included in the analysis

The second experimental series began by inserting a second three-way stopcock catheter (BD Connecta, 10 cm, BD Medical, USA) into the opposite pleural space through a mini-thoracotomy on the lateral left hemithorax. To simulate progressive HTX, blood was withdrawn from the arterial line, and 750 mL of blood was injected into the thorax in four steps using a 50 mL syringe (Omnifix, 50 mL, B. Braun Medical, Germany). Measurements were taken at the baseline level after completing the mini-thoracotomy and after four consecutive sets of blood volumes were injected, as shown in Table 1. Finally, to simulate a progredient HPTX, consecutive amounts of air were inserted into the left-sided HTX. The intrapleural air volume was increased to a maximum of 1500 mL of air (HPTX1500) but was stopped earlier if the animal did not haemodynamically tolerate this. In these cases, the last stable stage was included in the analysis. To compensate for blood loss and to stabilize circulation, an intravenous bolus of crystalloids was given.

### The wearable microwave instrument

**Fig. 1:**
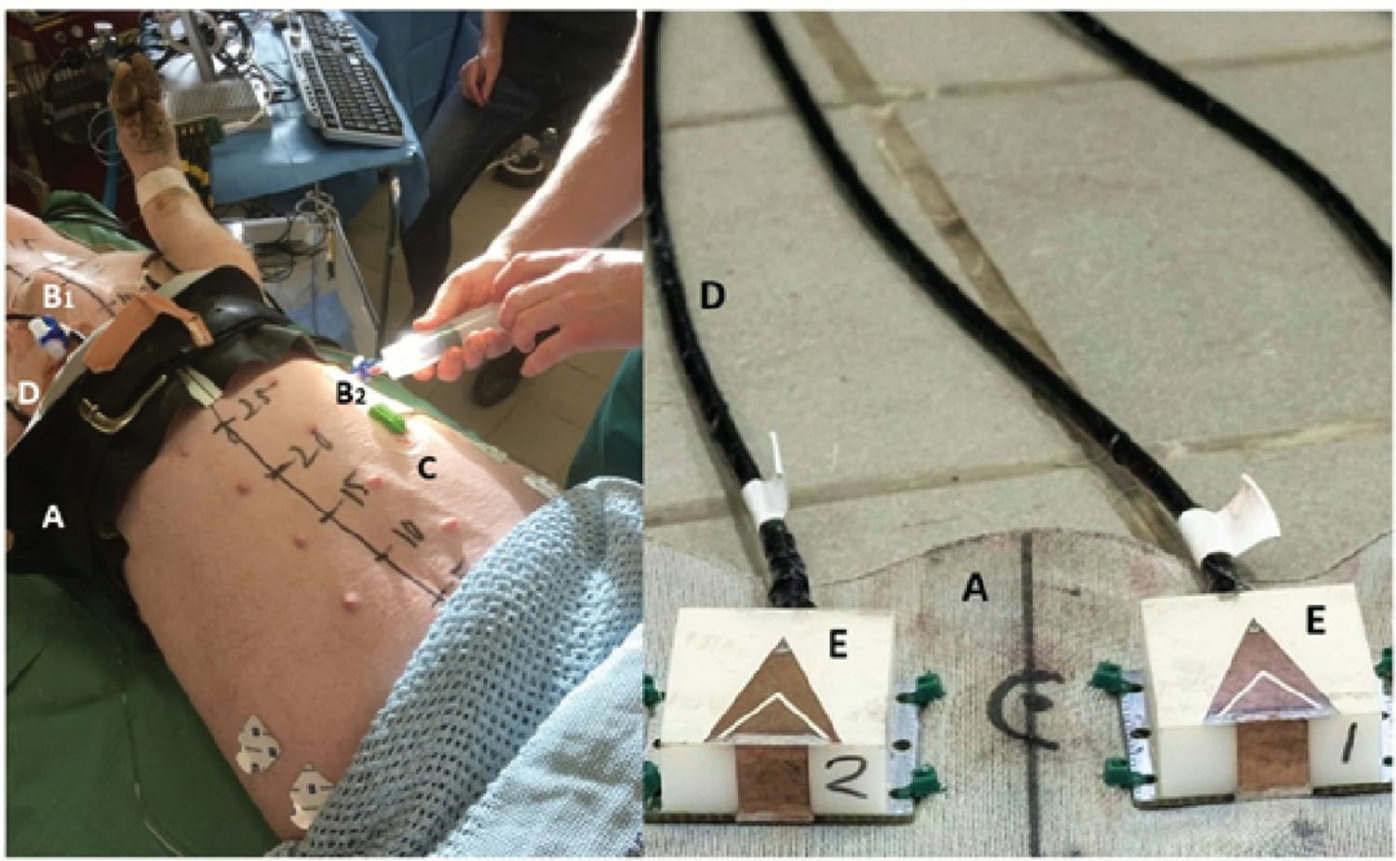
Experimental study setup. Left Porcine model with a microwave belt (A) around the thorax, the catheters inserted into the pleural cavity on the right side (B1) and left side (B2), ECG cable (C), and microwave cables (D). Right Image of two of the microwave antennas (E) inside of the microwave belt (A)

**Fig. 2:**
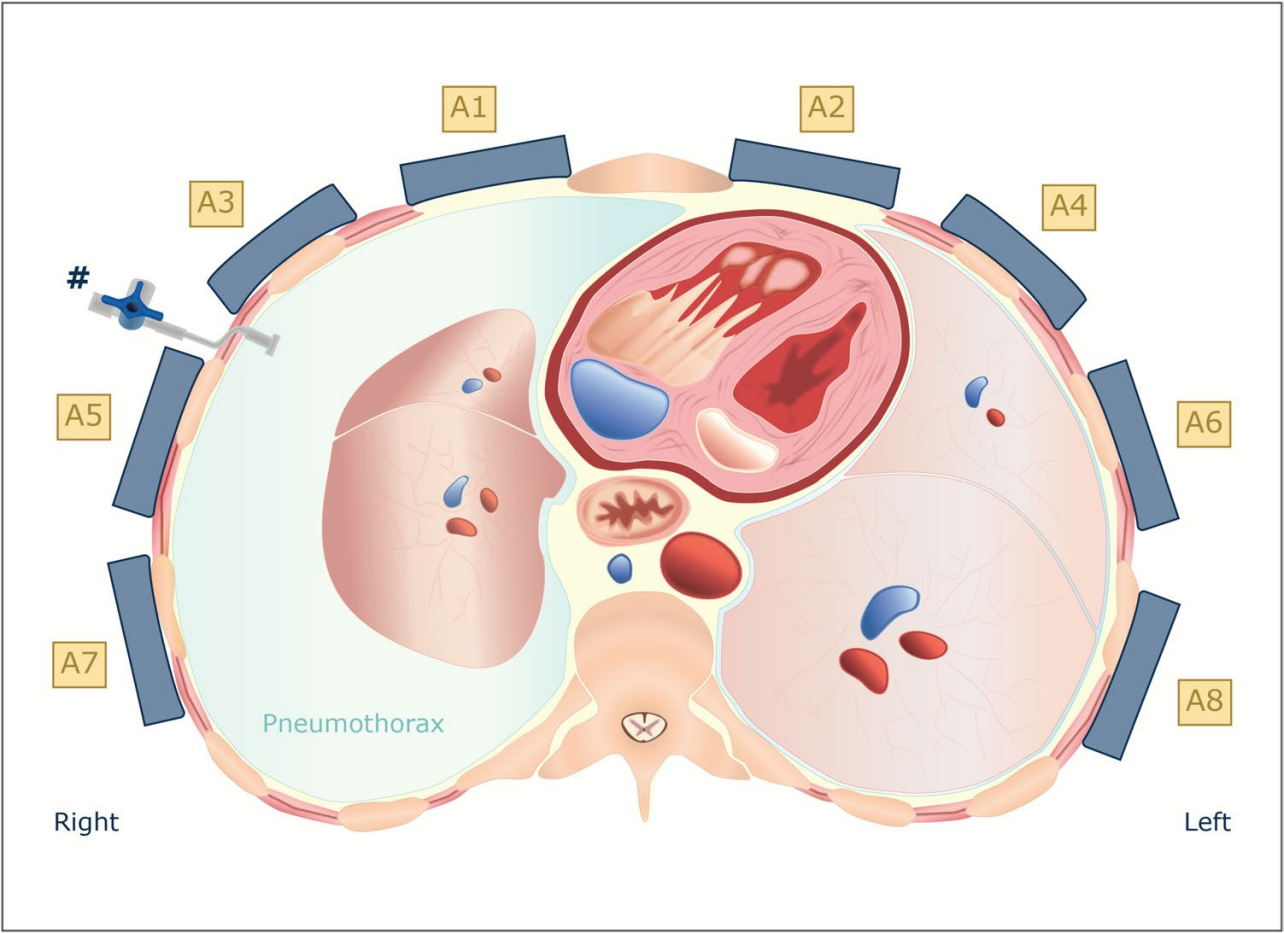
Geometrical arrangement of the eight antennas incorporated in the wearable microwave belt. The antennas were labelled so that the even-numbered antennas were located on the left side and the odd-numbered antennas were located on the right side. The image depicts a pneumothorax on the right side, induced by the insertion of air through the three-way stop cock (#), with collapse of the right lung

The experimental setup is shown in Fig. 1. The portable microwave device utilised eight antennas, as described by Trefna and Persson [27]. The configuration of these antennas is characterised by triangular microstrip plates, accompanied by a V-slot and a short-circuit wall measuring 37 × 25 × 14 mm. The antennas were originally developed for cranial microwave measurements, but were adapted in this study for circumferential measurements of the thorax. In order to facilitate this, the antennas were attached to a leather belt that was wrapped around the chest. The antennas were arranged as illustrated in Fig. 2 and connected to an eight-port semiconductor switching matrix, which in turn was connected to a network analyser [22]. We refrained from placing antennas at the back of the animal because such placement was deemed difficult to apply in a prehospital setting, where patients are generally placed in a supine position. The measurement procedure was automated, and all possible combinations of antenna pairs were measured. All hardware and software used for the microwave measurements were provided by Medfield Diagnostics AB (Gothenburg, Sweden).

### Measurements and data analysis

Baseline measurements were recorded after completing the min-thoracotomy with placement of the three-way stopcock on the right side and before induction of HTX on the left side. Microwave measurements were performed at each stage for the right-sided PTX (PTX50, PTX200, PTX500, PTX1000, and PTX1500), left-sided HTX (HTX50, HTX200, HTX500, and HTX750) and left-sided HPTX (HPTX50, HPTX200, HPTX500, HPTX1000, and HPTX1500).

As previously described [22], each microwave measurement involved measuring between all possible antennas, resulting in a total of 36 unique parameters. Of these parameters, 28 were defined as transmission parameters, where the transmitting and receiving antennas were different. The remaining 8 parameters were defined as reflection parameters, which occurred when the transmitting and receiving antennas were the same.

The microwave measurements were conducted in an electromagnetic frequency range of 0.1 to 2 GHz with a step size of 3.2 MHz. Each measurement formed a vector with more than 600 measurement points. The complex numbers (S-parameters) were analysed for changes in the magnitude and phase of the microwave signal.

To assess the reliability and stability of the microwave signal, each measurement was repeated ten times at each stage for PTX, HTX, and HPTX. The variance among these repeated measurements was analysed to determine the stability of the microwave signals.

To differentiate between the various states of PTX, HTX and HPTX from the baseline, a support vector machine (SVM) with linear kernel was utilised in this study. All classification tasks in this study were binary comparisons between each trauma condition and the baseline (e.g., PTX1500 vs. baseline, HTX750 vs. baseline), i.e. no multiclass classification was performed in this study. For each reflection coefficient, both magnitude and phase over the frequency range (0.1–2 GHz) were included as separate input features, while for each transmission coefficient, only magnitude was used. Prior to the training of the model, data smoothing was applied to all features to reduce noise.

In order to partition the data and prevent overfitting, a leave-one-out cross-validation (LOO) strategy was employed at the subject level. In each iteration of the experiment, the data from one pig was withheld from the training process, with the SVM being trained on the remaining nine subjects. Each subject contributed ten repeated measurements per class (trauma and baseline), resulting in twenty test samples per fold. The LOO scheme has been developed to ensure that each animal is used only once for testing purposes. In addition, it has been designed to preserve inter-individual variability while avoiding the need for a separate validation set. Diagnostic performance was assessed using classification accuracy, and the area under the curve (AUC) was calculated from the receiver operating characteristic (ROC) curve.

The data were tested for a normal distribution using the Anderson‒Darling test. As the data were not normally distributed, the single S-parameters were tested using a nonparametric test for dependent samples (Wilcoxon test). The analysis of “all parameters”, “all transmissions” and “all reflections” was conducted using paired t tests due to the high number of samples (n = 80–360). For each S-parameter, the data at all frequencies were averaged, resulting in a single value per S-parameter. *p* < 0.05 was considered to indicate statistical significance.

## Results

All ten animals were measured as described. However, only eight pigs could be analysed at the stage HPTX1500, as two pigs were not haemodynamically tolerating a haemopneumothorax volume of 750 mL of blood combined with 1500 mL of air. Consequently, the HPTX had to be relieved for this two pigs before the microwave measurements could be completed.

**Fig. 3:**
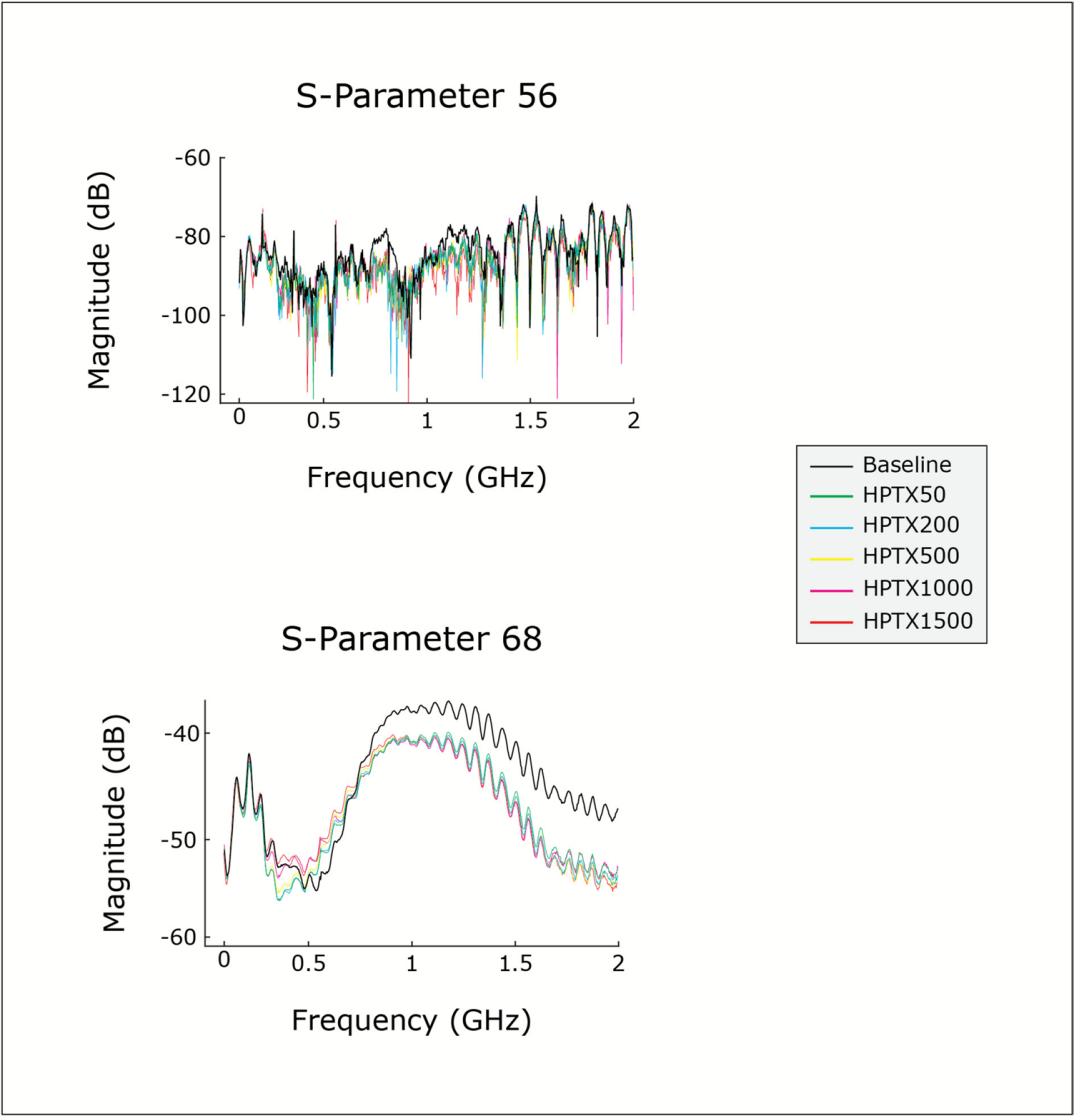
The average attenuation of the microwave signal for pig 1 over the frequency range from 0.1 to 2 GHz is presented as the mean value of the 10 repetitive microwave measurements. The data is shown for the transmission parameters 56 and 68 for the baseline and all HPTX stages. The antenna combination A5 and A6 (S-parameter 56) represents a parameter with a higher distance, which exhibits a high attenuation of the signal (−80 to −120 dB) and a high variance over the entire frequency range. The S-parameter 68 is an example of an antenna combination (A6 and A8) with a short distance and more stable signal, which exhibits a lower attenuation (−40 to −60 dB) and a typically lower variance, especially in the higher frequency range

**Fig. 4:**
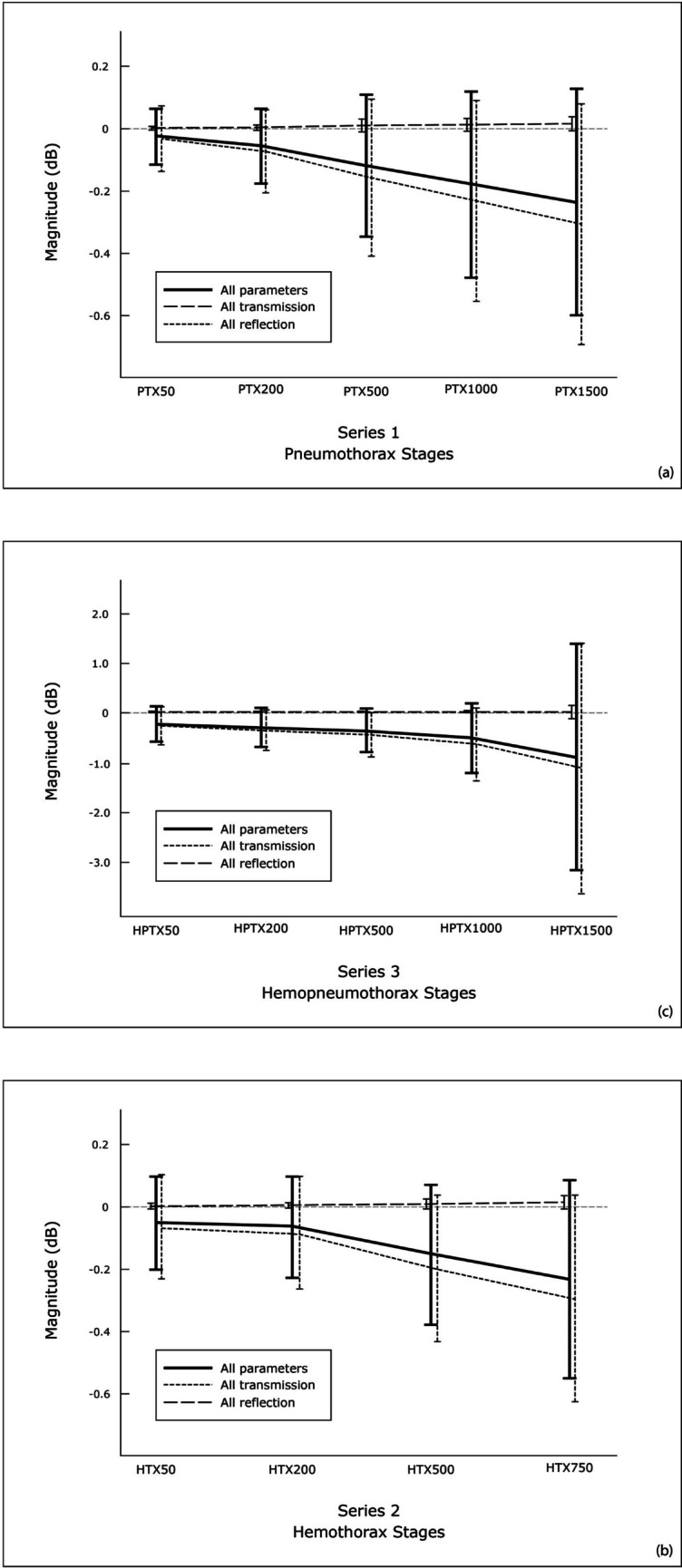
The plot represents the average change in magnitude for all repetitive measurements and pigs for all PTX (**a**), HTX (**b**) and HPTX (**c**) stages relative to baseline. The change in magnitude is presented as the mean ± SD for the sum of all parameters (“all parameters”, n = 36), the sum of all transmission parameters (“all transmissions”, n = 28) or the sum of all reflection parameters (“all reflections”, n = 8). For the “all parameters” and “all transmissions” group, the changes from baseline were found to be statistically significant for all stages in all series (PTX, HTX and HPTX) (p < 0.001). The transmission parameters demonstrated a clear increase in attenuation with increasing trauma stage, in contrast to the summed reflection coefficients. In addition to the attenuation of the magnitude, the variance of the measurement also increased with increasing trauma stage. The attenuation observed in the HPTX series (**c**) was found to be up to 10 times higher than that observed in the PTX and HTX series

This study examined the microwave signal to identify any changes in magnitude (i.e., the S-parameter). As the microwave signal passed through the thorax, it exhibited an attenuation in magnitude, expressed as negative decibel (dB) values, as shown in Figs. 3 and 4 and supplement material.

### Variance of the microwave signal

The baseline measurements indicated that the variance of the ten repeated measurements at baseline was dependent on the magnitude of the microwave signals. The magnitude of attenuation depended on the distance between the antennas and the frequency of the signal. It was found that longer distances and lower frequencies resulted in greater attenuation of the signal and greater variance. Consequently, the variance of the repeated measurements was up to 10% of the average value between the distant-placed antennas (high attenuation of the signals, approximately −70 dB), while signals from adjacent antennas (low attenuation of the signals, approximately −40 dB) were considerably more stable, with a low average variance of 1%.

For isolated PTX and HTX, the lower volume stages showed little variance in the repeated microwave measurements. However, larger volumes of isolated air or blood resulted in greater variance. This effect was even more pronounced for all of the HPTX stages and increased notably for the most severe trauma stage (HPTX1500), as shown in Fig. 3. There was no significant difference in variance between the transmission and reflection parameters.

### Interindividual variability

In addition to the variance between repeated measurements, the analysis of the magnitude of microwave signals between the baseline values for each animal showed high interindividual variability between the animals, with a range of 35.1 dB and an IQR of 18.6 dB.

### Changes in the magnitude

Statistically significant attenuation of microwave signals was observed following the induction of all stages of PTX, HTX and HPTX compared to the baseline measurements. This attenuation was observed when the parameters were aggregated to encompass either all transmissions, all reflections or all parameters, as illustrated in Fig. 4.

Only a few of the 36 S-parameters (e.g., individual antenna combinations) exhibited a significant change in magnitude for PTX50 (7 from 36), PTX 200 (16 from 36), HTX50 (10 from 36) and HTX200 (15 from 36). Notably, the antenna combinations closest to the accepted distribution of PTX (e.g. anterior S-parameters: S22, S24, and S26) exhibited significant attenuation at 50 mL of air. The injection of 50 mL of blood into the thorax (HTX50) resulted in significant alterations in the left-sided dorso-basal parameters (S18, S28, S37, S38, S48, S66, S77, S478, S88). In the higher volume series for PTX and HPTX, 70–80% of the single S-parameters exhibited significant changes. For HPTX50 to HPTX500, 77–94% of the S-parameters were significantly altered. While the combined parameters for the largest HPTX volumes (HPTX1000 and HPTX1500) were significant, only a few of the single-antenna combinations were significantly altered.

Overall, above a frequency of 0.6 GHz, the magnitude of the transmission parameters decreased for all the measurements. As previously stated, the reduction was more pronounced with increasing volumes for PTX, HTX, and HPTX. HPTX exhibited the largest decrease in magnitude, with a maximum of −8.7 dB in HPTX1500, while PTX1500 had a maximal decrease of −1.5 dB and HTX750 a maximal decrease of −1.9 dB.

However, the induction of trauma on one side of the thorax altered all transmission parameters. This was even evident for the antenna pairs on the opposite side of the injury. The induction of PTX on the right side resulted in attenuation of the transmission parameters on the left side, and conversely, the left-sided HTX and HPTX attenuated the transmission parameters on the right side.

The reflection parameters (e.g., S88, S77, S66, and S22) showed a pattern comparable to that of the transmission parameters. However, some of the reflection parameters demonstrated greater sensitivity to the lower trauma stages. Parameters S22 and S66 exhibited significant changes in response to PTX50 and PTX200, while S88 and S77 demonstrated significant attenuation in response to HTX50 and HTX200.

### Classification accuracy and ROC curve

The performance of the SVM classification was tested for each parameter and three different groups of parameters: ‘all parameters’, ‘all transmissions’, and ‘all reflections’, as shown in Table 2. The primary classification analysis concentrated on the most substantial injury levels (PTX1500, HTX750, HPTX1500), which represented the most distinct and clinically relevant signal changes. However, the SVM classification analysis for lower trauma grades showed poor accuracy in terms of correct classification.

**Table 2:** Support vector machine (SVM) classification accuracy

	SVM classification accuracy		
	PTX1500	HTX750	HPTX1500
All parameters	53	70	90
All transmissions	50	57	65
All reflections	56	60	85

Classification accuracy. SVM classification accuracy between the baseline and the highest level of PTX, HTX and HPTX (PTX1500, HTX750 and HPTX1500). The accuracy is shown as the % for all 36 parameters, all 28 transmission parameters and all 8 reflection parameters as input data to the SVM. For PTX1500, the highest accuracy was reached by using the reflection parameters as the input data, and for HTX750 and HPTX, by using all parameters as the input data

**Fig. 5:**
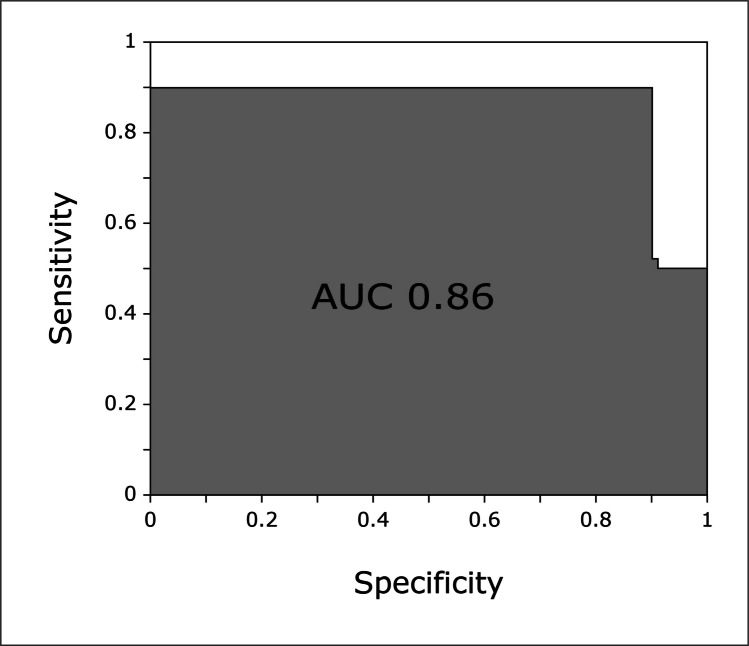
ROC curve with an AUC of 0.86 for the best SVM classifier between HPTX1500 and baseline (i.e., all 36 parameters used as the input data)

The highest accuracy in the classification analysis was achieved for HPTX. When all 36 parameters were used as input data for the SVM, 90% of the observations had their class correctly predicted for HPTX1500. The ROC curve in Fig. 5 shows that the best SVM model had an AUC of 86%. Both the sensitivity and specificity of the best SVM model for distinguishing HPTX1500 from baseline were 90%. For PTX and HTX, the classification accuracy was lower. According to this SVM model, the classification accuracy between the baseline and HTX750 was 70%. For isolated PTX, the SVM achieved the highest accuracy when using all eight reflection parameters as input data. The classification accuracy between the baseline and PTX1500 was 56%.

## Discussion

We modified a previously described porcine model of pneumothorax [28] to simulate severe chest injury involving three different types of injuries: PTX, HTX and HPTX. Oveland et al. demonstrated the suitability of this model for simulating various sizes of pneumothorax [25, 26]. However, the expansion of the porcine PTX model to include HTX and HPTX has not been previously reported. Despite the induced blood loss and the presence of significant amounts of blood and air in the thorax, almost all measurements were successfully completed without any accidental animal death.

The three injury types were chosen to replicate the severity and common occurrence of chest injuries after major trauma. We simulated progressively more severe injuries by increasing the volumes of PTX, HTX, and HPTX, ranging from subclinical to life-threatening conditions. This allowed us to evaluate microwave technology in different clinical scenarios in this porcine model.

### Detection of air

The results of this study demonstrate that microwave signals are significantly attenuated by free air in the chest. Even a small PTX, which may be challenging to diagnose using a supine CXR or US [26], can alter microwave signals. In particular, our findings indicate that a lower threshold of air is sufficient to cause significant changes in microwave signals for the combined parameters and for some of the single S-parameters. Low-volume PTXs that are undetectable via CXR but can be detected via CT are referred to as occult PTXs [29, 30]. Although not all occult PTXs require treatment, monitoring the progression of occult PTXs with repeated CXR or CT is recommended. Here, microwave technology may offer a clinically relevant advantage in the diagnosis of even very small volumes of free air around the lung. Furthermore, our results showed that an increasing PTX size also increases the attenuation of microwave signals. This suggests the potential of MWT not only to detect free air in the chest (e.g., PTX) but also to monitor the progression of the PTX size, e.g., during prehospital transport.

### Detection of blood

Our results show that isolated HTX significantly attenuates the combined microwave signals as well as single microwave signals when measured in proximity to the anticipated distribution of blood within the pleural cavity. This finding is relevant for the detection of an occult HTX, which is again defined as blood inside the pleural cavity that is missed by supine CXR but found on CT scans. Previous studies have shown that volumes of less than 500 mL of blood can only be accurately detected by CT [14, 28, 31] Although HTX volumes less than 500 mL, and in some cases up to 1000 mL, may not be visible on CXR, chest drainage is recommended for all sizes of HTX [14].

Our experiment demonstrated that microwave signals are significantly altered for low-volume HTX and across all stages of HPTX. This suggests that MWT could be a viable alternative diagnostic method for detecting occult HTX and has the potential to expand diagnostic options and bridge the gap between CT and CXR, with the added benefits of being transportable and noninvasive. Our findings supplement those of other studies on the clinical use of MWT. One example is the research from Candefjord et al., which demonstrated that MWT can detect free blood in the abdomen in a similar manner. Additionally, several studies have shown promising results in using MWT to detect intracranial bleeding [17–19].

### Detection of both air and blood

Regarding the detection of HPTX, we observed significant changes in microwave signals for all stages tested. As with PTX and HTX, the microwave measurements showed increasing attenuation as the volume of HPTX increased. The microwave signal changes were more pronounced for all HPTX measurements compared to those for isolated PTX and HTX, reflecting the ability of MWT to detect and alarm in the presence of severe trauma.

The infusion of 750 mL of blood into the left thorax during the HPTX series represents a clinically significant amount of haemorrhage. However, this amount of blood is in the diagnostic grey zone of occult HTX, which cannot be easily detected by clinical examination or CXR. Notably, the distinct changes in microwave signals observed during simulated HPTX also correlated with the high sensitivity and specificity achieved by the support vector machine (SVM) for HPTX1500.

### Diagnostic performance

In our pig model and using an SVM machine learning algorithm, HPTX could be classified most accurately. By using all 36 parameters as input data for the SVM classifier, 90% of the HPTX1500 measurements could be correctly classified. This results in a sensitivity and specificity for the microwave belt of 90% for the detection of HPTX, which is above the described diagnostic accuracy for US and CXR [15, 16]. However, this high degree of accuracy was only achieved for a pronounced HPTX (HPTX1500). Typically, an HPTX of this size can also be reliably diagnosed with CXR and US.

The diagnostic performance of our wearable microwave belt prototype in detecting isolated traumatic injuries (PTX or HTX) was lower (i.e., a diagnostic accuracy of 56% and 70%, respectively). In this proof-of-concept study, lower trauma levels for HPTX, PTX and HTX did not produce favourable SVM classification results due to the small sample size and limited input data. Noise and artefacts also contributed to the high variability of signal changes. These problems could be solved by improving the antennas and antenna array.

### Interindividual variance

When comparing the microwave signals between individual animals, a very high interindividual variance of the baseline measurements was observed. The interindividual variance far exceeded the changes in microwave signals induced by PTX, HTX and HPTX. This high interindividual variance could be explained by the different anatomy of the animals (size, thorax shape, etc.) and by the fact that our prototype and the microwave antennas used for this experiment were not specifically adapted to the anatomical features of a pig’s thorax and the position of the antennas was not fixed.

### Intraindividual variability

The variance of the ten repeated measurements for each microwave measurement showed mostly small variation, but some measurements and certain frequencies had a variance of up to 10%. The variance in the short-range measurements between adjacent antennas could be explained by the crosstalk phenomenon. Previous experiments have shown that close antennas are particularly prone to crosstalk, and these observations have also been reported for abdominal microwave measurements [17, 22]. The variance in the long-distance measurements between more remote antennas could be explained by respiratory movements and the lack of adaptation of the prototype to the thorax.

### Limitations

There are several limitations to our study that should be acknowledged. The induction of large volumes of PTX, HTX and HPTX can lead to significant changes in anatomy and physiology, including a shift of the mediastinum towards the uninjured side. These changes due to the induced trauma are highly individual and difficult to predict. As a result, two animals in our study were unable to tolerate HPTX1500. These individual changes in anatomy and physiology may also lead to differences in the attenuation of microwave signals. We observed an increase in signal variance within the higher stages of PTX, HTX and HPTX (Supplement material: Figures S1, S2 and S3).

The apparent sensitivity of microwave signals to changes in intrathoracic conditions was supported by the fact that trauma on one side of the thorax also resulted in altered microwave signals on the other side. Although a systematic spatial analysis of antenna signals was not carried out, this should be included in future work, as it could contribute to a more accurate understanding of the spatial sensitivity of microwave signals and which antenna placements that are most effective for detecting and monitoring injuries. Another limitation is that our study was conducted on a relatively small number of animals. Therefore, our results provide only a first indication of the potential of microwave technology and warrant further research. Typically, an SVM achieves greater accuracy when working with hundreds of individual datasets, but due to the limited number of animals, we did not have enough data to achieve optimal diagnostic accuracy for the stages other than HPTX1500.

In addition, the early version of the prototype used in our study may have influenced the stability of the microwave signals. We observed high individual variability and, in some cases, significant variance between the ten repeated measurements. The stability of the measurements could be improved by adapting the microwave device to the thoracic anatomy and improving the antenna design to reduce the crosstalk phenomena. Exploring alternative measuring instrumentations as well as antenna and array configurations could be a valuable area of future research, as it has the potential to improve the diagnostic capabilities of microwave technology in cases of traumatic chest and abdominal injuries {Candefjord, 2021 #296}. However, this falls beyond the scope of the present proof-of-concept study. Future research could consider integrating vital parameter monitoring, such as respiratory and heart rates, into the same hardware to expand the potential usage of MWT [23, 24]. Finally, our study was conducted in an animal model, namely, pigs, which have anatomical characteristics that may differ from those of humans. While our model successfully simulated PTX, HTX and HPTX, we were unable to determine the distribution of air or blood in the pleural cavity and, as a result, how precisely the microwave belt was positioned in relation to the injected air or blood inside the chest. In further research, controlling the distribution and volume of air and blood in the thoracic cavity using CT scans, as well as optimizing the positioning of the microwave device, may improve the performance of MWT.

## Conclusion

This is the first study to evaluate the potential of a portable microwave device for the diagnosis of traumatic chest injuries in a porcine model. The presence of air and blood, both isolated and combined, in the pleural cavity significantly altered the microwave signals in our porcine model. Moreover, the degree of attenuation correlated with the size of the PTX, HTX, and HPTX. Notably, the wearable microwave belt can detect signal changes even at air and blood levels that represent occult PTX and HTX. Nevertheless, further improvement of the technology is necessary in terms of antenna design and position. Prior to commencing human clinical trials, these enhancements must be validated in an optimised, CT-controlled experimental study.

## Supplementary Information

Below is the link to the electronic supplementary material.

Supplementary file1 (DOCX 1018 KB)

## Data Availability

The datasets used and/or analysed during the current study are available from the corresponding author on reasonable request.

## Abbreviations

AUC: Area under the curve
CT: Computed tomography
CXR: Chest X-ray
etCO2: End-expiratory CO2
FiO2: Inspiratory oxygen fraction
HPTX: Haematopneumothorax
HTX: Haematothorax
MWT: Microwave technology
PTX: Pneumothorax
ROC: Receiver operating characteristic
SVM: Support vector machine
US: Ultrasound
Vt: Tidal volume
