# Supplementary material for: Microwave technology for detecting traumatic chest injuries in a porcine model
Source: Med Biol Eng Comput. 2025 Dec 23;64(3):949–61. doi: 10.1007/s11517-025-03495-8 (PMC13061816; doi:10.1007/s11517-025-03495-8)
Supplement: Supplementary file 1 — Supplementary file1 (DOCX 1018 KB) [file 11517_2025_3495_MOESM1_ESM.docx]

**Supplementary Information**

**Microwave technology for detecting traumatic chest injuries in a porcine model**

Philipp Seidel^1, 2^, Nils Petter Oveland^2, 3^, Marianne Oropeza‐Moe^4^, Linh Nguyen^5^, Andreas Fhager^5^, Mikael Persson^5^, Mikael Elam^6^ & Stefan Candefjord^5,7^

^1^Department of Intensive Care Medicine, Stavanger University Hospital, Stavanger, Norway

^2^Department of Quality and Health Technology, Faculty of Health Sciences, University of Stavanger, Stavanger, Norway.

^3^Department of Anesthesiology, Stavanger University Hospital, Stavanger, Norway.

^4^Department of Production Animal Clinical Sciences, Faculty of Veterinary Medicine, Norwegian University of Life Sciences, Sandnes, Norway.

^5^Department of Electrical Engineering, Chalmers University of Technology, Gothenburg, Sweden.

^6^Department of Clinical Neurophysiology, Sahlgrenska University Hospital, Gothenburg, Sweden.

^7^SAFER Vehicle and Traffic Safety Centre at Chalmers, Gothenburg, Sweden.

Corresponding author: Philipp Seidel, philipp.seidel@uis.no, (+47) 48505574

***Figure S1.*** *The relative change in magnitude from the baseline for all S-parameters, for all repetitive measurements and pigs, is shown for the PTX stages. The changes in magnitude (dB) are represented as the mean ± SD. Blue indicates antenna pairs on the right side and red indicates antenna pairs on the left side of the thorax. Green indicates antenna pairs measured across the thorax.*

**
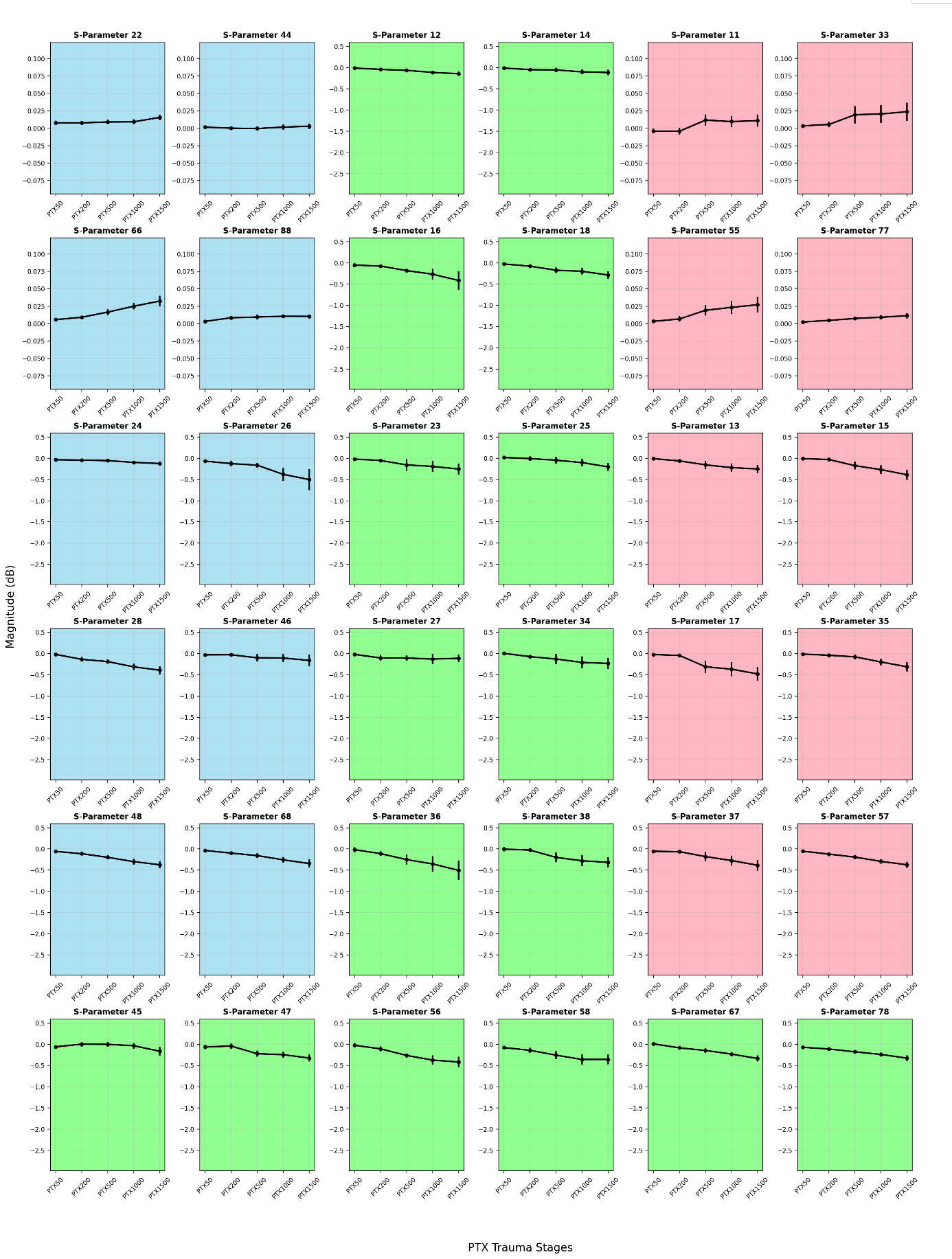
**

***Figure S2.*** *The relative change in magnitude from the baseline for all S-parameters, for all repetitive measurements and pigs, is shown for the HTX stages. The changes in magnitude (dB) are represented as the mean ± SD. Blue indicates antenna pairs on the right side and red indicates antenna pairs on the left side of the thorax. Green indicates antenna pairs measured across the thorax.*

*
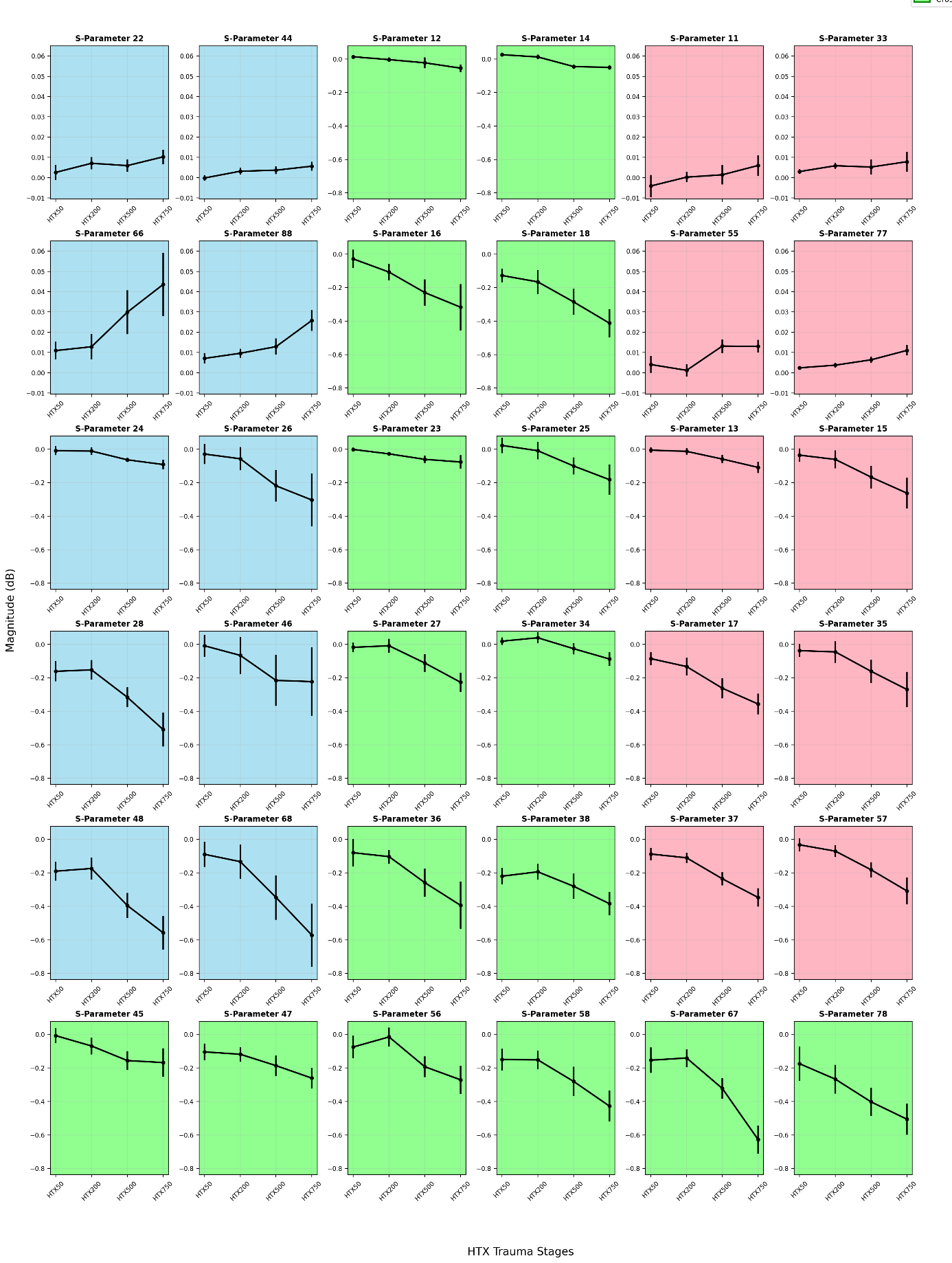
*

***Figure S3.*** *The relative change in magnitude from the baseline for all S-parameters, for all repetitive measurements and pigs, is shown for the HPTX stages. The changes in magnitude (dB) are represented as the mean ± SD. Blue indicates antenna pairs on the right side and red indicates antenna pairs on the left side of the thorax. Green indicates antenna pairs measured across the thorax.*

**
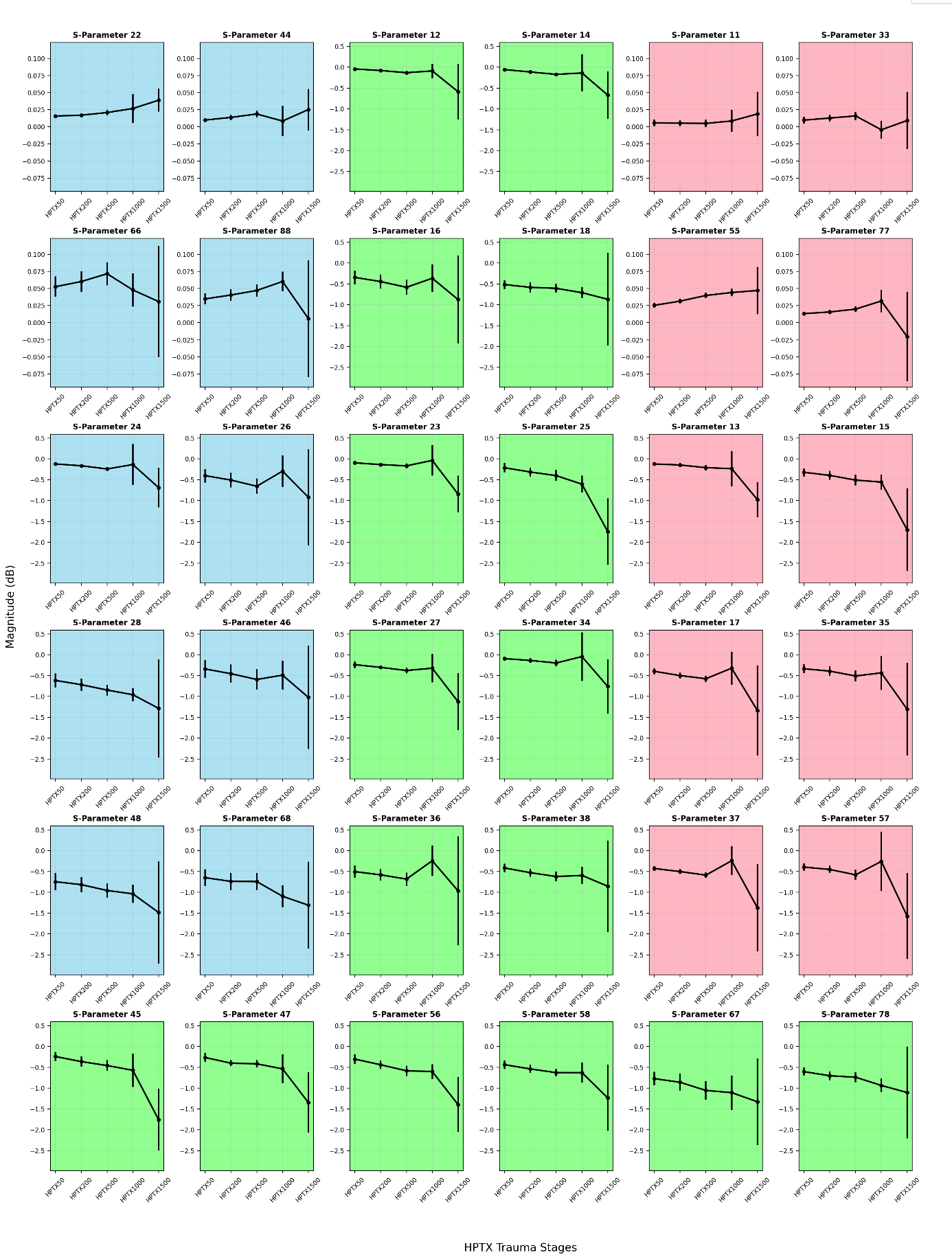
**
